# Correspondence

**Published:** 1871-03

**Authors:** 


### CORRESPONDENCE.

MARCH 1, 1871.

*Drs. Powell & Goldsmith.—Gentlemen:*—The second number of your valuable journal has been received, and after a careful perusal of its pages, justice compels me to say, that in my humble judgment, it contains more that is valuable—more that is constantly needed by the every day practitioner, than any number of any medical journal, of sixty-four pages, that I ever had the pleasure to read. The plan, in all of its departments, is admirable, and must meet, to the fullest extent, the most sanguine expectations of all its readers. I am proud of the high position you have taken in defence of "Medical Ethics"—the "cap-stone" of the profession—the greatest security and protection to the afflicted.

But a short time since, I visited a sick lady, in company with a medical friend. A few moments after we entered her room, there came in a singular looking man, and inquired if he had been sent for. Upon being answered in the affirmative, my medical friend asked the gentleman whether he was a regular doctor, to which he replied, "I am not; I have no *diplomy*; Heaven has given me the power to heal all diseases. I use no medicine. I am a *nateral* doctor, one after Christ's plan—the laying on of hands." He also stated that he had been sent for to treat one of our most intelligent and respectable citizens—a Christian gentleman. What a libel upon the Bible!

I am not ignorant of the fact that medical ethics requires every member of the profession to expose all such imposters, and some of us in this section are very much inclined, and have almost determined to make the effort, and would, if they were certain the Georgia Medical Association would sustain them. Will it do it? If the Georgia Medical Association would carry into practical operation the report made—I think by Dr. McDowell and others, in 1869—neither the people or the profession would be troubled very long with either home or itinerant *Quacks.* We hope the Association will do something, at its next meeting, with a view to instructing the people on the evils of quackery and secret remedies. Justice, alike to the people and the profession, demands the good and true men of the profession to be silent no longer, but to unseal their lips, buckle on their armour, and meet the monster, "*Quackery*"—the popular and legally-authorized poisoner—the great enemy to scienee, in his chosen avenues, as has been recommended by Prof. G. B. Wood, of New York, and Prof. Bache, of Philadelphia. Like your correspondent * * * my battle in the practice will soon end—a few years, at best, and I must retire. I have a great desire, before I die, to see every worthy member of the profession united and working in harmony under "right organization—organization that is sustained, clung to, abided by—organization that will read out and condemn those who are faithless to it, and sustain those who are right, without fear, favor or affection."

My son, a young man, is just acquiring a good practice, and if I believed the profession would abandon its principles, which we have all been taught to revere, I should at once advise him to leave it. And I think if something is not done, the good material of the country will devote its talent and energies in other more honorable and lucrative pursuits. Wishing you much success, and hoping the best for our cause and science,

I am yours, truly, W. A. N

---

*Drs. Powell & Goldsmith.—Gentlemen :*—I thank you for not publishing my letter. On reflection, I am satisfied that some of my allusions were too personal, and that your course is the proper one—not to admit anything of a personal nature into your journal. But as you allow me the privilege of discussing or stating, in the COMPANION, what, in my judgment should or should not be tolerated by the profession, I will, therefore, state a few things :

1st. The members of the profession should exercise more circumspection in receiving young men as students in their offices. Good moral character and adequate literary attainments, should be indispensable qualifications, before any physician should allow a student to commence the study of medicine in his office. 2d. Medical Colleges should be required to be uniform in their curricula: to require their students to have studied medicine three years under a private preceptor—should have a *real* attendance on the part of the student during their sessions—should have a complete corps of professors, and lastly, to make the final examination for the Doctorate as rigid as possible. They should require their graduates to read, and conform their professional action to the Code of Ethics. And they should distinctly state, and reserve the right, when graduating a class, that any violations of the ethics, or dishonorable or unprofessional conduct on their part, would invariably be followed by a withdrawal of their diplomas.

I suggest these things because of the necessity which exists. Many are surrounded by legalized quacks—men violating every principle of the ethics—but who go unrebuked by the institutions from which they graduated, or by the profession at large. Unless something is done—unless the good men of the profession will unite as one man, and put an end to this condition of things, the profession will sink to rise no more. Will not some of the able medical men of Georgia go to the next meeting of the Georgia Medical Asssciation, with well-digested plans, and recommend some action to be taken in this direction? Men cannot afford to leave their homes and go to the Association, to hear read long essays upon science, when it refuses to take notice of the dishonorable and irregular conduct of its members, and the cause of science injured by its non-action. Harmony cannot be established, so long as the wiry, selfish medical demagogue is placed upon terms of equality in the Association, with the good law-abiding physician, to thus receive the same advantage, support and privileges at home.

Some intelligent gentlemen, who can spare the time, should go to the Association with a plan for the organization of County Societies in every State of the Union, to be in harmony with that of the State and the National Associations. This matter ought to receive such consideration in the Association as to call for a Committee to recommend a plan, and earnestly request the medical gentlemen of every county in the State to form medical societies, to vindicate and uphold the laws of the profession, at every sacrifice and at all hazards.

It is to be hoped that tha American Medical Association, at its next meeting, will take some decided action by defining clearly what shall be required of Medical Colleges, to entitle them to the full and entire confidence of the profession. I am aware that our voluntary organizations have no legal right to alter college charters, or require their faculties to conform to medical law, but we have the right to say when they do not conform to law, that they should be cut off from the confidence, recognition and support of the profession ; and to deny their students or graduates private and public recognition. Until this is done, and Colleges are made to assume proper relations to the ethics and the profession, all over the country, there can be no harmony or advancement in our science.

Please excuse me for asking you to withhold my name, as I am " surrounded by the enemy" and cannot afford to put myself up as a target, when I stand alone without the actual or moral support of my professional brethren. T. O. C.

